# Co-Inoculation of an Endophytic and Arbuscular Mycorrhizal Fungus Improve Growth and Yield of *Helianthus tuberosus* L. under Field Condition

**DOI:** 10.3390/jof7110976

**Published:** 2021-11-17

**Authors:** Saranya Khaekhum, Jindarat Ekprasert, Thanapat Suebrasri, Wasan Seemakram, Wiyada Mongkolthanaruk, Nuntavun Riddech, Sanun Jogloy, Sophon Boonlue

**Affiliations:** 1 Department of Microbiology, Faculty of Science, Khon Kaen University, Khon Kaen 40002, Thailand; K.saranya@kkumail.com (S.K.); jindaek@kku.ac.th (J.E.); seemakram.w@gmail.com (W.S.); wiymon@kku.ac.th (W.M.); nunrid@kku.ac.th (N.R.); 2 Faculty of Medical Science, Nakhon Ratchasima College, Nakhon Ratchasima 30000, Thailand; s.thanapat@kkumail.com; 3 Department of Agronomy, Faculty of Agriculture, Khon Kaen University, Khon Kaen 40002, Thailand; sanun@kku.ac.th

**Keywords:** arbuscular mycorrhizal fungi, biofertilizer, *Exserohilum rostratum*, endophytic fungi, plant growth promoter, sunchoke

## Abstract

Endophytic fungi (EPF) and arbuscular mycorrhizal fungi (AMF) symbioses can promote the growth and productivity of several types of plants. This work aimed to investigate the effect of co-inoculation of an EPF *Exserohilum rostratum* NMS1.5 and an AMF *Glomus etunicatum* UDCN52867 g.5 on the growth and yields of sunchoke (*Helianthus tuberosus* L.) compared to the effects of full-dose and half-dose chemical fertilizer (15–15–15) under field conditions. Several plant growth parameters of the co-inoculated plants were significantly higher than the other treatments. Remarkably, such an effect was relatively equal to that of the full-dose chemical fertilizers. Moreover, the co-inoculation of EPF and AMF significantly improved the tuber yield production, even better than the use of a chemical fertilizer. This is the first report to show that plant growth promoting effects of the co-inoculation of EPF and AMF were exceptionally greater than those of the chemical fertilizer. Therefore, our EPF and AMF could potentially be used as a biofertilizer for promoting the growth and yield of sunchoke in the fields.

## 1. Introduction

*Helianthus tuberosus* L. (Jerusalem artichoke or sunchoke) is a native plant to North America, which belongs to the *Asteraceae* family. Its tubers contain inulin, a naturally occurring polysaccharide beneficial to human and animal health [1]. The tubers can be used as raw materials to produce healthy food products, animal feed additives, and bioethanol [2]. Due to its great potential for food and industrial production, sunchoke has been widely cultivated and consumed in Thailand [3]. However, to promote the growth and yield of sunchoke, farmers usually use chemical fertilizers, which often leads to an accumulation of toxic residues in the area [4]. It is therefore important to find alternative ways to upscale the production of sunchoke without causing harm to the environment. 

Plant growth-promoting fungi (PGPF) are ubiquitous fungi symbiotically associated with plants such as endophytic fungi (EPF) and arbuscular mycorrhizal fungi (AMF) [5,6,7]. These fungi can promote the growth of their host plants and also protect the plants from diseases [4,8,9,10]. EPF are micro-fungi that internally infect living plant tissues without causing any disease to the plants [11]. They can provide multiple benefits to their host plants such as promoting plant hormone production, phosphate solubilization, tolerance to abiotic and biotic stresses, and antimicrobial agents against plant pathogens [12]. AMF, biotrophic endophytic microorganisms belonging to the phylum Glomeromycota, are a group of fungi that form symbiotic associations within plant roots [13]. Similar to EPF, AMF can help host plants to increase growth and productivity by enhancing the uptake of water and mineral nutrients, improving the soil quality, enhancing stress tolerance, and protecting against plant pathogens [14,15]. However, the distinct feature of the AMF that distinguishes them from EPF is their ability to colonize the belowground parts of plants, which was restricted to plant roots, while AMF fungi only colonized the roots. Some EPF are also capable of colonizing the aboveground parts of plants such as leaves, stems, flowers, and seeds [16,17].

Our previous studies showed that a single inoculation of either EPF strain *Exserohilum rostratum* NMS1.5 [8] or AMF species *Glomus etunicatum* UDCN52867 g5 [6] could promote the growth of sunchoke in pot trials under greenhouse conditions. We then selected these two fungi for further study on their plant growth promoting effects on sunchoke under field conditions. Several studies have reported the co-inoculation of EPF and AMF to promote plant growth, for example, Xu et al. [18] found that synergistic interaction between EPF and AMF could simultaneously promote plant growth and enhance drought resistance in peanut plants. Moreover, Wezowicz et al. [10] reported that the co-inoculation of AMF and EPF could help the *Verbascum lychnitis* seedlings to survive and improve various plant growth parameters, especially the biomass and yield of the plants. Nevertheless, the effects of the interaction of EPF and AMF on enhancing the growth and production of sunchoke under field conditions has not yet been examined. This work then aimed to focus on the effects of co-inoculation between an EPF strain *Exserohilum rostratum* NMS1.5 and an AMF strain *Glomus etunicatum* UDCN52867 g5 on the growth and yield of sunchoke cultivated under field conditions. The findings of this study will be applied for the production of bio-fertilizers and become a standard practice for sunchoke growers in the future.

## 2. Materials and Methods

### 2.1. Endophytic Fungi Inoculum Preparation 

The endophytic fungus used in this study was *Exserohilum rostratum* NMS1.5 (NCBI accession number LC565443), which enhances the growth and yield of sunchoke cultivated in pot trials under greenhouse conditions [8]. Strain NMS1.5 was inoculated onto potato dextrose agar (PDA) and incubated for seven days. Fresh mycelia were subsequently transferred to sterilized sorghum seeds and incubated under static conditions until full colonization appeared [19]. After 14 days of incubation, sorghum seeds with full fungal colonization were used as an inoculum for plant growth experiments. 

### 2.2. In Vitro Effect of Endophytic Fungi on AMF Spore Germination

The stock culture of AMF strain *Glomus etunicatum* UDCN52867 g.5 (NCBI accession number LC564865) was obtained from the Mycotechnology Laboratory at the Department of Microbiology, Khon Kaen University. The germination of AMF spores was tested using a sandwich technique [20]. Briefly, AMF spores were surface-sterilized using Chloramine-T solution (2% *w/v*) for 5 min. Then, the spores were rinsed with sterile distilled water three times. Afterward, fifty surface-sterilized spores were placed between two sheets of sterile filter membrane (Millipore Corporation, Bedford, United States, 50 mm diameter, 0.45 µm pore diameter), which were moistened with 1 mL of EPF supernatant, and then transferred into sterile soil in sterile Petri dishes saturated with sterile distilled water. The established “sandwich of spores” was covered with plastic frame slides and incubated in the dark at room temperature for four weeks prior to the germination test. AMF spore germination was observed under a stereoscopic microscope (Nikon SMZ445, Ningbo Shinea Imp. & Exp. Co., Ltd. Zhejiang, China). The percentage of germination was calculated according to the formula below:

(1) $$\%\;\mathrm{Germination}=\frac{\mathrm{Total}\;\mathrm{number}\;\mathrm{of}\;\mathrm{germinated}\;\mathrm{spores}}{\mathrm{Total}\;\mathrm{number}\;\mathrm{of}\;\mathrm{spores}}\times 100$$

### 2.3. AMF Inoculum Preparation 

AMF spores were multiplied using maize seeds (*Zea mays* L.) by the pot culture technique according to the method by Boonlue et al. [21]. Then, pots were placed under greenhouse conditions for three months. In order to observe AMF root colonization under a stereomicroscope, roots were stained with 0.05% trypan blue for 5 min, and destained with distilled water. The number of spores was calculated and percentage of root colonization was determined according to the method by Trouvelot et al. [22].

### 2.4. Preparation of Sunchoke Seedlings and Fungal Inoculation 

Sunchoke seedlings were prepared according to the method described by Sennoi et al. [3]. Tubers were cut into small pieces with 2–3 active buds each and pre-germinated in moist coconut coir dust. Four-day-old seedlings were transferred into plastic trays (one seedling per hole) filled with black rice husk mixed with soil (3:1 *w/w*). Two sorghum seeds infected with a EPF strain NMS1.5 were placed under the seedlings, prior to transplantation. The AMF inoculation of strain UDCN52867 g.5 with approximately 100 spores g^−1^ soil inoculum was added to the soil in the close proximity to the sunchoke roots. For the co-inoculation treatment, two sorghum seeds infected with an EPF and AMF inoculum (100 spores g^−1^ soil) were combined and then added in the same manner as previously described. Seedlings were grown in a greenhouse under ambient conditions for 14 days. Healthy seedlings with two true leaves developed were transplanted into field plots.

### 2.5. Soil Physicochemical Analysis

Soil samples in the experimental field were collected for the determination of physicochemical properties (Table 1). Soil pH was determined in a soil suspension (soil: water ratio of 1:1 (*w/v*)) using a pH meter (Becthai Bangkok Equipment and Chemical, Thailand). Soil organic matter was evaluated using the Walkley-Black chromic acid wet oxidation method [23]. Total nitrogen (N) was extracted according to the micro-Kjeldahl nitrogen method [24], followed by a measurement of indophenol blue formation by the Berthelot reaction [25]. Total phosphorus (P) and potassium (K) were extracted from soil samples according to the wet oxidation digestion method [26] using a mixed nitric acid and perchloric acid at a ratio of 2:1 (*v/v*). Colorimetric analysis of total P was carried out using the molybdenum blue method [26] by measuring an absorbance at a wavelength of 820 nm using a spectrophotometer (Thermo Fisher Scientific Inc., Tokyo, Japan). Total K was analyzed by a flame photometer at a wavelength of 768 nm [26]. Available P content in soil was extracted according to the Bray II method [27], and then quantified by the molybdenum blue colorimetric method [28]. 

### 2.6. Experimental Design and Treatments

The field experiment was carried out at an agronomy farm, Khon Kaen University, Khon Kaen, Thailand. Initially, soils were plowed three times using conventional tillage equipment. A field trial was arranged as a randomized complete block design (RCBD) with four replications within a plot size of 15 m^2^ (width 3 m × length 5 m), in which each plot consisted of five rows having a length of 5 m with a space between each row of 0.5 m. Plant spacing between each row was 0.5 m and 0.4 m between each plant within the same row. Six treatments included: non-inoculated control plants (T1); a full dose of chemical fertilizers (N–P–K of 15–15–15) (T2); a half dose (50%) of chemical fertilizers (N–P–K of 15–15–15) (T3); a single inoculation of EPF (*E. rostratum* NMS1.5) (T4); a single inoculation of AMF (*Gl. etunicatum* UDCN52867 g.5) (T5); and a co-inoculation of *E. rostratum* NMS1.5 and *Gl. etunicatum* UDCN52867 g.5 (T6). Each sunchoke seedling was transferred into a 5 cm-deep hole. In treatments T4–T6, AMF inoculum approximately 2000 spores g^−1^ soil inoculum per seedling and/or 4 sorghum seeds infected with EPF was placed beneath the seedling roots. In the cases of treatments T2 and T3, a chemical fertilizer was added at a designated dose after 30 days of transplantation. In order to maintain moisture for plants, water was applied daily for 30 min using a mini water sprinkler. Weeding was carried out once a week after transplanting. At 90 days after transplantation, plants were collected for further analysis.

### 2.7. Determination of Plant Growth Parameters

At 90 days after planting, six plants per treatment of each plot were collected for analysis. Plant growth parameters including plant height, stem diameter, SPAD chlorophyll of leaf, and leaf area index were determined. The SPAD chlorophyll values of plant leaves were measured using a chlorophyll meter SPAD-502 plus. Leaf area was measured using a leaf area meter (Li-3100C Area meter). In order to quantify leaf chlorophyll content, fresh leaves were cleaned thoroughly and ground. Then, 1 g of ground leaves was used for chlorophyll extraction using 80% acetone. The mixture was centrifuged at 4000 rpm for 10 min in order to retrieve the supernatant. Chlorophyll contents in the samples were determined by measuring the absorbance at a wavelength of 663 nm (Abs663) and 645 nm (Abs645) using a spectrophotometer (Hitachi High-Tech Science Corporation, Tokyo, Japan). Chlorophyll a, chlorophyll b, and total chlorophyll contents were calculated according to Arnon [29] following the equations below:

Chlorophyll a content = (12.7 × Abs_663_) − (2.69 × Abs_645_)

Chlorophyll b content = (22.9 × Abs_645_) − (4.68 × Abs_663_)

Total chlorophyll content = (20.2 × Abs_645_) + (8.02 × Abs_663_)

Fresh leaves, stem and roots were dried in an oven at 80 °C for three days prior to determination of plant biomass including fresh and dry weight of leaves, stem, and roots. The parameters determining the quality of plant roots including length, diameter, surface area, and volume were evaluated by scanning the root samples using an Epson scanner V800 PHOTO, and the data were analyzed using WINRHIZO Pro2004a software (REGENT Instruments Inc., Quebec, QC, Canada).

### 2.8. Determination of Nutrient Uptake by Plants 

Plant uptake of nutrients including nitrogen (N), phosphorus (P), and potassium (K) was determined in dried plant samples. Total N was extracted from the samples by the micro-Kjeldahl nitrogen method, then the content was measured using Auto-Analyzer 3 (AA3), SEAL Analytical, Germany; Method No. G–253–00 Rev.1 (Multitest MT7/MT8, SEAL Analytical GmbH, Norderstedt, Germany) at an absorbance of 660 nm [30]. Total P was extracted following a wet oxidation method using a mixed nitric acid and perchloric acid at a ratio of 2:1 (*v/v*) [31]. Then, total P content was determined following the molybdovanadate with acid persulfate digestion method using a spectrophotometer at a wavelength of 420 nm [31]. Total K was extracted following a wet oxidation method using a mixed nitric acid and perchloric acid at a ratio of 2:1 (*v/v*) [31]. Subsequently, total K content was quantified by flame photometer at a wavelength of 768 nm (Flame photometer, Model 410 Sherwood, UK) [26]. 

### 2.9. Determination of Tuber Yield, Yield Components, and Harvest Index

At the harvest stage (120 days after planting), tubers were harvested and washed before assessing yield qualities including fresh weight of individual tubers, weight of tubers per plant, number of tubers per plant, and inulin content. The tuber inulin contents were analyzed according to the method described by Saengkanuk et al. [32]. Four mature plants in each plot were sampled and used for the determination of yield components, which were number of tubers per plant and tuber size. The number of tubers per plant was manually counted. Fresh tubers were washed and then air-dried in order to determine their fresh weights. The number of tubers per plant were determined from nine plants in each plot. Harvest index was calculated using the formula as follows:

(2) $$\;\mathrm{Harvest}\;\mathrm{index}\;\left(\mathrm{HI}\right)=\frac{\mathrm{Dry}\;\mathrm{weight}\;\mathrm{of}\;\mathrm{tubers}\;\left(g\right)}{\mathrm{Total}\;\mathrm{dry}\;\mathrm{weight}\;\mathrm{of}\;\mathrm{plants}\;\left(g\right)}\;$$

### 2.10. AMF and EPF Colonization Assessment 

Roots associated with AMF were stained with trypan blue following a modified method described by Koske and Gemma [33]. To prepare the roots for staining, roots were washed under running tap water to remove soil particles. Then, the cleaned roots were boiled in 2.5% KOH solution at 90 °C for 10 min in order to clear the roots for further observation. Roots were rinsed with excess water to remove the KOH solution. Then, roots were immersed in 1% HCl solution at room temperature overnight followed by rinsing with water. The staining was carried out using 0.5% trypan blue in acetic glycerin solution prior to colonization assessment. Roots were cut into 1 cm fragments, mounted on glass slides, and the samples were soaked with lactophenol. AMF structures and their root colonization were observed using a light microscope (Nikon Eclipse50i, Nikon Corporation, Tokyo, Japan) at 40–100× magnification. Observations of EPF presented in the roots were carried out in a similar manner to the AMF colonization assessment. Root colonization was observed in 30 root fragments from different zones of the roots per treatment. The percentage of AMF colonization was evaluated according to the method described by Trouvelot et al. [22]. Colonization frequency of EPF in roots was determined according to the method described by Mehmood et al. [34] using the formula as follows:

(3) $$\;\%\;\mathrm{Colonization}\;\mathrm{frequency}=\frac{\mathrm{Total}\;\mathrm{number}\;\mathrm{of}\;\mathrm{root}\;\mathrm{colonized}}{\mathrm{Total}\;\mathrm{number}\;\mathrm{of}\;\mathrm{roots}}\times 100$$

### 2.11. Statistical Analysis

All statistical analyses were performed using a Statistix 10.0 software program. Data were analyzed according to a randomized complete block design (RCBD) from four plot replications. The data were subjected to an analysis of variance (ANOVA). The least significant difference (LSD) test was applied to test the significant difference among the treatment means at *p* ≤ 0.05.

## 3. Results

### 3.1. In Vitro Effects of Endophytic Fungi on AMF Spore Germination and AMF Inoculum Preparation

Effects of the endophytic fungus *E. rostratum* NMS1.5 on spore germination of an AMF strain *Gl. etunicatum* UDCN52867 g.5 were investigated using a sandwich assay technique in order to ensure the non-antagonistic effects between these fungal strains. The results showed that AMF spores could germinate in the presence of the cell–free supernatant of the EPF at a germination rate of as high as 67%. This indicated that EPF did not exhibit detrimental effects on the AMF spore germination, and thus implied an absence of antagonistic effects between our selected EPF and an AMF. Therefore, an EPF strain *E. rostratum* NMS1.5 and the AMF strain *Gl. etunicatum* UDCN52867 g.5 were used in further experiments under field conditions. The multiplication of AMF spores in maize (*Zea mays* L.) showed a colonization rate of 63% and the number of spores of 46 spores g^−1^ dry soil. This suggested successful AMF spore multiplication for use in field conditions.

### 3.2. Colonization of Endophytic Fungi and Arbuscular Mycorrhizal Fungi in Sunchoke

**Figure 1:**
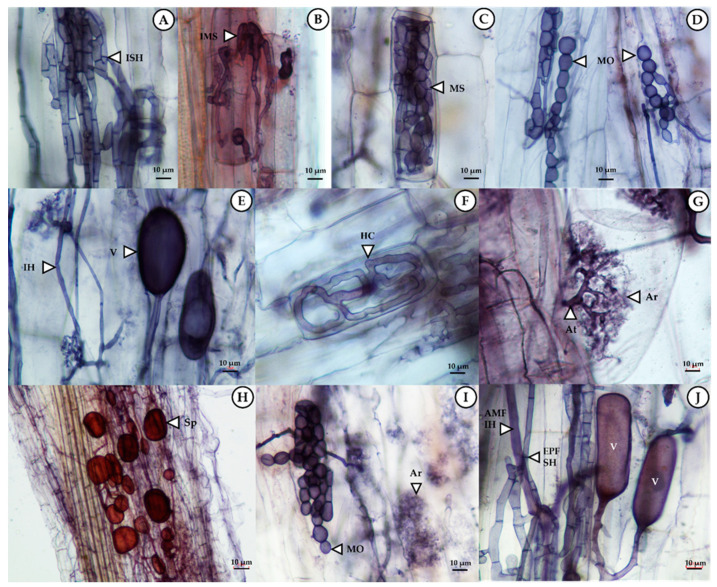
Colonization structures of endophytic fungi: EPF (**A**–**D**), arbuscular mycorrhizal fungi: AMF (**E**–**H**), and co-inoculation with EPF and AMF (**I**–**J**) in the roots of sunchoke observable in EPF-inoculated plants treatment (T4), AMF-inoculated plant treatment (T5), and co-inoculation of EPF and AMF-treated plant treatment (T6), respectively. (**A**) Intracellular septate hyphal: ISH, (**B**) Initiation, development, and formation of microsclerotia: IMS, (**C**) Microsclerotia: MS, (**D**) Moniliform cell: MO, (**E**) Intracellular hyphal: IH and Vesicles: V, (**F**) Hyphal coil: HC, (**G**) Arbuscule trunks: At and Arbuscules: Ar, (**H**) AMF spores: Sp, (**I**) Moniliform cell of endophytic fungi: MO, Arbuscules of AMF: Ar, and (**J**) AMF intracellular hyphal: AMF IH, AMF Vesicles: V, and Intracellular septate hyphal of endophytic fungi: EPF SH. Scale bars are 10 µm.

**Figure 2:**
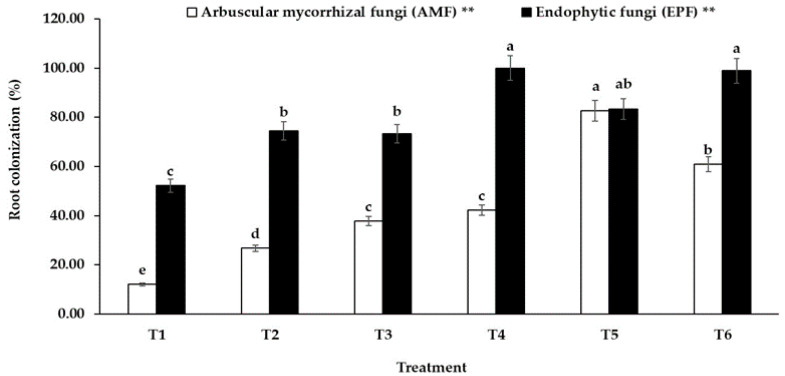
Root colonization (%) of AMF and EPF in sunchoke roots at 90 days after planting. Treatments are indicated as follows: (T1) uninoculated plants, (T2) full-dose chemical fertilizer-treated plants, (T3) half-dose chemical fertilizer-treated plants, (T4) EPF-inoculated plants, (T5) AMF-inoculated plants, and (T6) co-inoculation of EPF and AMF-treated plants. Error bars represent standard deviations of four replicate data. Different letters above the bars indicate significant differences. ** Significant difference at *p* ≤ 0.01.

Successful colonization of an endophytic fungus *E. rostratum* NMS1.5 and an AMF strain *Gl. etunicatum* UDCN52867 g.5 in sunchoke plants were investigated at 90 days after transplantation. Moreover, the structures of both EPF and AMF colonization in the roots of sunchoke are shown in Figure 1. The percentage of colonization of both fungi in sunchoke roots under different treatments is also presented. Figure 2 reveals the colonization of both EPF and AMF present in all treatments including the uninoculated control. This was because sunchoke plants were grown in an open field where soils were not sterile and all environmental factors were uncontrollable, which resembled the actual practice carried out by local sunchoke growers. 

However, in terms of AMF and EPF colonization, all treatments showed a significantly higher colonization than that in the uninoculated plants. The highest AMF colonization was found in the AMF-inoculated plants (T5) as expected. It appears that AMF colonization in the co-inoculated plants (T6) was lower than the colonization in the plants treated with AMF alone (T5). Moreover, the application of chemical fertilizer either full dose or half dose could significantly enhance AMF colonization in sunchoke. The half-dose chemical fertilizer-treated plants (T3) showed a higher percentage of root colonization than that found in the full-dose chemical fertilizer-treated plants (T2). Accordingly, the colonization potential of AMF decreases with an increase in chemical fertilizer application, suggesting that a full dose of chemical fertilizer had an adverse effect on AMF colonization. In terms of EPF colonization, the EPF-inoculated plants (T4) had the highest percentage of EPF root colonization, which was not significantly different from that in the co-inoculated plants (T6), indicating successful colonization of EPF regardless of the presence of AMF. Interestingly, EPF colonization in the AMF-inoculated plants (T5) was not significantly different from that in the full-dose chemical fertilizer-treated plants (T2) and the half-dose chemical fertilizer-treated plants (T3). 

### 3.3. Effects of co-inoculation of EPF and AMF on Plant Growth Performance of Sunchoke

There were two growth stages of sunchoke plants: the growing stage and the harvest stage. During the first stage, the dry masses of leaves and stems continued to increase and peaked at 15 to 17 weeks after planting. The dry biomass was then gradually decreased afterward. During the harvest stage, which is from 18 to 20 weeks after planting, the tubers rapidly expanded and the number of tubers and biomass also increased [35]. However, the sunchoke growth period varied depending on the climate conditions and sunchoke variety [36]. In this study, plant growth parameters were measured at 90 days after planting, while the harvested tuber yields were evaluated at 120 days after planting. Plant growth parameters including plant height, stem diameter, SPAD chlorophyll values, leaf area, and plant biomass (leaf, stem, and root) at 90 days after planting were presented in Table 2. The results showed that all plant growth parameters of the plants inoculated with either AMF alone, EPF alone, or mixed culture of AMF and EPF significantly increased when compared to the uninoculated plants (the control). Moreover, most of these qualities of the inoculated plants, especially when co-inoculated with AMF and EPF, were comparable to those of the plants applied with full-dose chemical fertilizer, and much better than those of the half-dose fertilizer applied plants. In this regard, the quality of plant leaves including SPAD, leaf area, and leaf biomass were significantly improved by co-inoculation of AMF and EPF up to the relatively same range as those of the full-dose chemical fertilizer applied plants. The results also indicated that co-inoculation of AMF and EPF could positively affect stem and root biomass similar to full-dose fertilizer. This suggested that co-inoculation of an AMF strain *Gl. etunicatum* UDCN52867 g.5 and an EPF strain *E. rostratum* NMS1.5 could effectively enhance the plant growth parameters of sunchoke, and its effects were even as high as the effects of full-dose chemical fertilizer.

Table 3 presents the plant root growth in terms of length, volume, diameter, surface area, and nutrient uptake of sunchoke. The results revealed that all of the plant root growth parameters were the highest in plants co-inoculated with EPF and AMF, which were significantly higher than the uninoculated control plants and even higher than the full-dose chemical fertilizer-treated plants for some parameters. Moreover, plants inoculated with AMF had plant root quality (length, surface area, diameter, and volume) similar to those of the plants co-inoculated with EPF and AMF, which was significantly higher than that of plants in the other treatments. Interestingly, most of the root growth parameters of the plants treated with chemical fertilizer were not significantly different from those of the control plants. N uptake was not significantly different among treatments, while *p* and K uptake was significantly higher in plants treated with AMF. Additionally, plants co-inoculated with EPF and AMF had a significant increase in P uptake at a relatively similar level as that of the AMF-treated plants, whereas chemical fertilizer had no significant effect on nutrient uptake. In summary, AMF could facilitate P and K uptake in sunchoke and had a stronger positive influence on many root parameters than the chemical fertilizer.

### 3.4. Effects of Co-Inoculation of EPF and AMF on Chlorophyll Contents 

**Figure 3:**
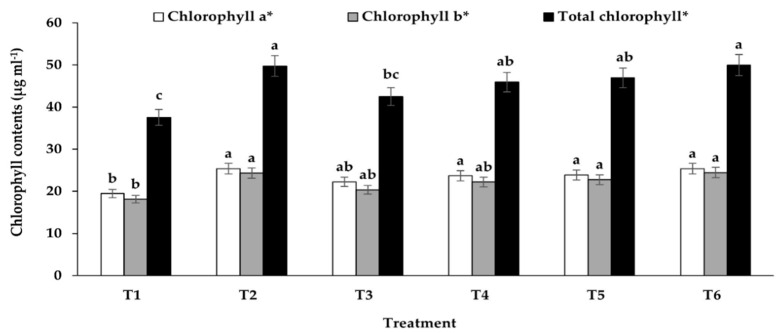
Chlorophyll a, b, and total chlorophyll contents in sunchoke. Data are the means of four replications. Six treatments are indicated as follows: (T1) uninoculated plants, (T2) full-dose chemical fertilizer-treated plants, (T3) half-dose chemical fertilizer-treated plants, (T4) EPF-inoculated plants, (T5) AMF-inoculated plants, and (T6) co-inoculation of EPF and AMF-treated plants. Error bars represent the standard deviations of four replicate data. Different letters above the bars indicate significant differences. * Significant difference at *p* ≤ 0.05.

The results in Figure 3 show that the highest chlorophyll a, b, and total chlorophyll contents were found in plants co-inoculated with EPF and AMF, which was not significantly different from those of the plants treated with full-dose chemical fertilizer. Similar to the results of other plant growth parameters, it was found that the use of half-dose chemical fertilizer could not significantly enhance chlorophyll contents in sunchoke when compared to those of the control plants. Furthermore, the inoculation of plant growth promoting fungi of sunchoke plants, either a single inoculation of EPF or AMF or a co-inoculation of both EPF and AMF, could increase the chlorophyll a, b, and total chlorophyll contents. 

### 3.5. Effects of co-inoculation of EPF and AMF on the Yield of Sunchoke

In order to investigate the effects of the co-inoculation of EPF and AMF on the yield of sunchoke, yield components including tuber yield, number of tubers, tuber size, harvest index, and tuber inulin contents were determined at the harvest stage (120 days after planting). The results presented in Table 4 indicated that all yield productions of sunchoke plants treated with co-inoculation with both EPF and AMF were the highest among those of other treatments including the ones with chemical fertilizer. Interestingly, while the number of tubers per plant, weight of each tuber, and weight of tubers per plant were significantly increased in plants treated with either EPF, AMF, or co-inoculation of both fungi, no significant change in these parameters in the plants treated with chemical fertilizer, regardless of a full or half-dose application when compared to the control plants. This suggested that the application of chemical fertilizer barely improved the yield of sunchoke, but plant growth promoting fungi, which were EPF, AMF, and both of them together, could effectively enhance sunchoke yield. Moreover, the highest harvest index was found in plants co-inoculated with both EPF and AMF. The effects of EPF or AMF alone on the harvest index were comparable to the effects of full-dose chemical fertilizer. This indicates that to enhance the sunchoke yield, the application of chemical fertilizer can be replaced by the use of the plant growth promoting fungi in our work. Interestingly, it was obvious that % inulin content in sunchoke tubers increased only when the plants were treated with either one of our fungi or both of them, but not with chemical fertilizer. All of these results imply that the inoculation of EPF and AMF in our work as well as their co-inoculation was more beneficial in improving the yield of sunchoke under field conditions than the use of the chemical fertilizer.

## 4. Discussion

In our previous studies, an EPF strain *E. rostratum* NMS1.5 and an AMF strain *Gl. etunicatum* UDCN52867 g.5 competently enhanced the growth and yield of sunchoke in pot trials under greenhouse conditions [6,8]. Although a single inoculation of either EPF or AMF had a significant increase in sunchoke yield, plants treated with the co-inoculation of both fungi could improve the highest sunchoke yields, suggesting efficient plant growth promotion due to the synergistic effect of EPF and AMF. This work aimed to investigate the effects of co-inoculation on sunchoke under field conditions. 

Our findings showed that all plant growth parameters in plants treated with co-inoculation of EPF and AMF (T6) were significantly higher than those of the uninoculated plants. Moreover, co-inoculated plants also had higher chlorophyll a, b, and total chlorophyll concentrations, which were not significantly different from those found in the full-dose chemical fertilizer-treated plants (T2). This suggested the potential use of both fungi as biofertilizers to enhance sunchoke growth instead of the application of chemical fertilizers. These findings agreed with Rahimi et al. [37], who showed that the application of biofertilizer was more efficient than chemical fertilizers in improving plant growth. Although the application of chemical fertilizers could increase plant growth during the initial stage, there have not been any long-term studies to ensure its sustainability. This suggests that we should not rely on the PGP microorganisms native to cultivation regions for promoting plant growth. In addition, the selection of EPF and AMF species to produce biofertilizers is necessary. Therefore, the application of our EPF and AMF strains is important in improving sunchoke growth and yield.

We found that the plant growth promoting ability of an AMF strain UDCN52867 g.5 under field conditions followed similar trends to those when studied under pot trials under greenhouse conditions in our previous works. For example, this present study showed that a single inoculum of our AMF strain UDCN52867 g.5 could increase chlorophyll a, b, total chlorophyll content, and plant uptake of P and K (Table 3). These results were in agreement with the study in pot trials by Nacoon et al. [6], which reported that the AMF could increase K uptake in sunchoke plants. However, this AMF strain had better performance in promoting plant growth in the field than in the pots. This might be because AMF probably needs an undisturbed environment where it originated to interact effectively with plants. Surprisingly, there have only been a few studies comparing the effects of either AMF or EPF colonization under greenhouse and field conditions. Copetta et al. [38] found that AMF inoculation significantly affected melon growth, flower and fruit production, and fruit quality in both field and greenhouse conditions at different growth stages. Under the field conditions, AMF-inoculated plants flowered earlier than the uninoculated plants, whereas under the greenhouse conditions, all plants flowered at the same time. Furthermore, AMF inoculation also improved seed size and weight as well as increased seed germination and root elongation at the first stage of growth in the field. This could depend on different environmental conditions, especially temperature and light, which are the major influences for the establishment of symbiosis and plant responses [39]. Therefore, AMF inoculation significantly improved fruit quality and plant performance in the field. Wijesooriya and Deshappriya [40] found similar effects of EPF on the promotion of growth and yield of a traditional rice variety in Sri Lanka under greenhouse and field conditions. The study showed that during 10 weeks after planting, EPF inoculated plants had a better increase in plant height, fresh weight, and dry weight than the non-inoculated plants under both field and greenhouse conditions. At the harvesting stage (12 weeks), the results showed an increase in fresh weight and dry weight of the plants grown under the field condition when compared to those in plants grown under the greenhouse condition. Remarkably, EPF inoculation could better enhance biomass and rice yield under field conditions when compared with those grown under greenhouse conditions. Therefore, an inoculation of either AMF or EPF seems to function more efficiently under the field conditions than the greenhouse conditions. This is probably because agricultural fields are, in general, regularly disturbed by tillage, naturally-altered environmental conditions, and fertilization, all of which could help strengthen fungal adaptability and enhance fungal development in the fields more than when they were subjected to a non-disturbed environment under the greenhouse [41,42]. Moreover, under field conditions, the fungi could better expand its hyphal network and rapidly colonize plant roots, allowing them to occupy a large proportion of diverse neighboring roots. This affected the colonization, growth ability, and survival of other fungi that naturally occur around the roots [43,44].

Interestingly, although it is well-known that AMF can enhance plant root growth both vertically and horizontally [45], the results of our work showed that sunchoke roots could grow much better when an EPF strain NMS1.5 was co-inoculated. The effect was even higher than the effects by chemical fertilizer. This is likely because the EPF strain NMS1.5 can produce plant growth hormones, especially IAA and GA_3_ [8], which influence a range of developmental processes in plants such as cell elongation, cell division, and root initiation [46]. Ibiang et al. [47] reported the effect of co-inoculation with AMF and endophytic fungus *Penicillium pinophilum* EU0013 on the growth and root colonization of tomato and lettuce. The results showed that fungal root colonization and plant growth promotion such as plant height and total fresh weights (biomass) showed a higher increase in the co-inoculated plants than those in the plants inoculated with either AMF or EPF alone. Therefore, it seems that it is not only the effects of AMF itself in facilitating root growth, but also the hormones produced by EPF that additionally enhance the enlargement and elongation of sunchoke roots. Taking the benefits of AMF strain UDCN52867 g.5 and EPF strain NMS1.5 together, the most efficient way to apply microbial inoculum for promoting root growth, and thus the growth of sunchoke plants is the co-inoculation of AMF and EPF. 

According to the root colonization study of EPF and AMF, we found EPF and AMF colonization in all treatments, indicating the existence of indigenous EPF and AMF in the sunchoke roots. However, EPF colonization in the AMF-inoculated plants was not significantly different from that in the full and the half-dose chemical fertilizer-treated plants. This suggests a relatively similar effect of AMF and chemical fertilizer on EPF colonization, in which AMF might provide nutrients for EPF to grow and colonize plant roots. This assumption was also evidenced by a decrease in AMF colonization in the co-inoculated plants. Likewise, chemical fertilizer could act as a source of nutrients for EPF as can be seen by an increase in EPF colonization in T2 and T3 when compared to the control. In addition, in the treatment with EPF, AMF, and co-inoculation of EPF and AMF, the colonization of both fungi was significantly higher than the control and the chemical fertilizer treated plants. This implies the successful colonization of our EPF and AMF added in the experiment. Therefore, any alteration in the growth and yield of sunchoke plants that occurred at the harvest stage was a result of our EPF and AMF, and not mainly of the indigenous cultures in the field. Moreover, the results showed that the percentage of EPF colonization was higher than that of AMF in almost all treatments. The reasons for this could be explained by two different mechanisms. First, both EPF and AMF receive shelter and nutrients from their plant host, which might trade off in resource allocation between fungal symbionts. EPF may gain spatial and temporal priority, leaving fewer carbon reserves for AMF, and thereby might reduce the AMF colonization. In our experiment, we found EPF colonization in plant roots after two weeks of inoculations, while the colonization of AMF was rarely observed (data not shown), resulting in higher EPF than AMF colonization rates in co-inoculated roots. Another possible mechanism is that the bioactive compounds produced by endophytes such as alkaloids might have an inhibitory effect on AMF [48]. However, our in vitro study suggested that the cell-free supernatant of an EPF strain NMS1.5 did not inhibit spore germination by an AMF strain UDCN52867 g.5 (67% AMF spore germination). Therefore, the latter mechanism is unlikely the cause of inequivalent root colonization between EPF and AMF in our experiment. Nevertheless, the synergistic interaction between EPF and AMF has not yet been clearly elaborated. Liu et al. [12] suggested that the interactions between fungal endophytes and AMF may be species-specific, for example, *Gl. mosseae* and *Gl. intraradices* were much more sensitive to the presence of endophytes than *Gl. etunicatum* and *Gl. claroideum*. However, the results in our work contradicted the claim in which the fungal endophyte belonging to the genus *Exserohilum* and an AMF belongs to a different genus, *Glomus*, but they could still work well together in promoting the growth and yield of their plant host. We therefore suggest that there might also be other factors involved in the regulation of synergistic interaction between EPF and AMF in the environment.

In terms of sunchoke yield production, the results show that the effects of co-inoculation between *E. rostratum* NMS1.5 and *Gl. etunicatum* UDCN52867 g.5 outperformed the other treatments. We found that the tuber yield, number of tubers, tuber size, harvest index (HI), and tuber inulin contents of sunchoke significantly increased when the plants were co-inoculated with EPF and AMF. These yield parameters were comparable and even greater than those of the plants treated with the chemical fertilizer (Table 4). Based on our previous study in pot trials, the results suggest that the production of IAA and GA_3_ by an EPF strain NMS1.5 is possibly the main factor affecting the enhancement of tuber formation. In this regard, IAA plays an important role in tuber development, possibly by regulating tuber growth through mediating meristem identity, cell division, and the plane of cell division [49]. Meanwhile, GA_3_ acts as a stimulating agent for cell elongation for tuber formation, supporting the development of stolons and the growth of their tubers [50]. Likewise, AMF may play an important role in initiating and stimulating the production of tubers through hormonally–mediated processes [51]. It might be that, in our present work, EPF and AMF concomitantly alleviated tuber development both quantitatively and qualitatively. Note that this synergistic effect could exert sunchoke yield production in the field even better than the use of chemical fertilizers, which has been a common practice for sunchoke growers in this region. All of this evidence suggests that the co-inoculation of EPF strain NMS1.5 and AMF strain UDCN52867 g.5 can replace the application of chemical fertilizer for promoting the growth and yield of sunchoke. 

## 5. Conclusions

This work investigated the potential use of co-inoculation of EPF strain *E. rostratum* NMS1.5 and AMF strain *Gl. etunicatum* UDCN52867 g.5 to enhance the growth and yield of sunchoke under field conditions. The study showed that the co-inoculation of EPF and AMF in sunchoke plants could improve plant growth parameters including plant height, stem diameter, SPAD chlorophyll values, leaf area, and plant biomass much higher than those of the uninoculated plants. Interestingly, the plant growth promoting effects of the co-inoculation were relatively comparable to those of the application of a full-dose chemical fertilizer. In contrast, plants treated with a half dose of chemical fertilizer did not enhance the growth of sunchoke when compared to uninoculated plants. Furthermore, the use of a single inoculation of either EPF or AMF and the co-inoculation of EPF and AMF could promote sunchoke yield production, which are tuber yield, yield components (number of tubers and tuber size), harvest index (HI), and tuber inulin contents than those of the plants applied with chemical fertilizer. Particularly, the highest tuber yield production was found in the co-inoculated plants. This suggests that the co-inoculation of EPF and AMF is more beneficial to plants than a single inoculation of either one of them. Our work clearly demonstrates that the synergistic effects of EPF and AMF could promote the yield of sunchoke under field conditions. Note that this study is the first to show successful application of the co-inoculation of *E. rostratum* NMS1.5 and AMF *Gl. etunicatum* UDCN52867 g.5 for growth promotion and yield production of sunchoke, in which the effects were far better than the use of chemical fertilizer under field conditions. Therefore, the development of a bio-fertilizer containing both EPF and AMF for use in sunchoke fields is worth further study. 

## Figures and Tables

**Table 1:** Soil chemical characteristics and physical properties.

Physical Properties			Texture Class	Chemical Properties					
Sand (%)	Silt (%)	Clay (%)		pH (1:1 H_2_O)	Organic Matter (%)	Total N (mg kg^−1^)	Total P (mg kg^−1^)	Total K (mg kg^−1^)	Available P (mg kg^−1^)
89.93	7.93	2.14	Sand	5.37	0.589	0.0188	150.50	171.64	44.13

**Table 2:** Plant growth parameters of sunchoke including plant height, stem diameter, SPAD chlorophyll values, leaf area, and plant biomass (leaf, stem, and root) at 90 days after planting under field conditions.

Treatment	Plant Height (cm)	Stem Diameter (cm)	SPAD Values	Leaf Area Index	Plant Biomass (g/plant)		
					Leaf	Stem	Root
T1: Control (no inoculation)	102.75 ± 8.51 ^c^	0.83 ± 0.13 ^d^	38.08 ± 1.52 ^c^	1599.70 ± 466.52 ^b^	53.93 ± 3.99 ^c^	54.03 ± 15.19 ^c^	27.06 ± 4.45 ^b^
T2: Full dose of chemical fertilizers	125.90 ±8.98 ^a^	1.24 ± 0.09 ^a^	40.85 ± 0.61 ^a^	2817.90 ± 560.76 ^a^	94.24 ± 10.19 ^a^	94.34 ± 16.37 ^a^	41.94 ± 3.34 ^a^
T3: Half dose of chemical fertilizers	109.15 ± 2.25 ^bc^	1.03 ± 0.05 ^c^	39.29 ± 0.72 ^b^	2088.60 ± 360.30 ^ab^	66.53 ± 12.44 ^bc^	62.27 ± 15.48 ^bc^	37.82 ± 7.47 ^ab^
T4: EPF inoculation	113.12 ± 1.67 ^b^	1.11 ± 0.06 ^bc^	40.68 ± 0.14 ^a^	2 467.10 ± 521.52 ^ab^	74.36 ± 8.44 ^abc^	71.68 ± 8.43 ^abc^	40.83 ± 9.79 ^ab^
T5: AMF inoculation	115.33 ± 5.59 ^b^	1.14 ± 0.05 ^abc^	40.80 ± 0.20 ^a^	2333.80 ± 778.98 ^ab^	73.08 ± 14.26 ^abc^	78.24 ± 16.73 ^abc^	41.13 ± 12.03 ^a^
T6: co-inoculation of EPF and AMF	118.58 ± 4.34 ^ab^	1.18 ± 0.04 ^ab^	40.78 ± 0.25 ^a^	2581.60 ± 838.22 ^a^	83.47 ± 17.48 ^ab^	89.91 ± 15.46 ^ab^	42.36 ± 10.44 ^a^
CV	5.65	7.33	1.91	26.02	16.60	19.77	23.75
F-test	*	*	*	*	**	**	*

Different letters indicate significant differences among values within the same column analyzed by the LSD test. Values are means ± SD (*n* = 4). * Significant difference at *p* ≤ 0.05; ** Significant difference at *p* ≤ 0.01; ns, non–significant difference.

**Table 3:** Plant root growth parameters including length, volume, diameter, surface area, and nutrient uptake of sunchoke at 90 days after planting under field conditions.

Treatment	Plant Root Growth				Nutrient Uptake (g kg^−1^)		
	Length (cm)	Volume (cm^3^)	Diameter (mm)	Surface Area (cm^2^)	Nitrogen (N)	Phosphorus (P)	Potassium (K)
T1: Control (no inoculation)	2794.20 ± 873.21 ^c^	20.43 ± 9.36 ^b^	1.03 ± 0.56 ^b^	1355.90 ± 280.53 ^c^	1 0.83 ± 2.10	1.88 ± 0.43 ^b^	20.3 ± 1.53 ^b^
T2: Full dose of chemical fertilizers	4899.20 ± 713.88 ^ab^	31.06 ± 1.05 ^ab^	1.50 ± 0.31 ^ab^	2292.60 ± 465.73 ^ab^	1 3.30 ± 0.86	2.65 ± 0.17 ^ab^	23.75 ± 4.61 ^ab^
T3: Half dose of chemical fertilizers	3525.30 ± 1166.25 ^bc^	25.90 ± 4.53 ^ab^	1.51 ± 0.34 ^ab^	1647.80 ± 355.24 ^bc^	1 2.15 ± 1.77	2.38 ± 0.66 ^ab^	21.73 ± 3.20 ^ab^
T4: EPF inoculation	4906.60 ± 962.55 ^ab^	33.10 ± 7.31 ^a^	1.81 ± 0.31 ^a^	2376.70 ± 572.39 ^ab^	1 1.45 ± 1.98	2.88 ± 0.48 ^a^	22.05 ± 1.86 ^ab^
T5: AMF inoculation	5573.50 ± 454.77 ^a^	33.33 ± 6.54 ^a^	1.86 ± 0.66 ^a^	2501.00 ± 819.05 ^a^	1 2.63 ± 4.09	3.15 ± 0.58 ^a^	24.73 ± 3.71 ^a^
T6: co-inoculation of EPF and AMF	5664.70 ± 1925.18 ^a^	35.25 ± 7.49 ^a^	2.03 ± 0.36 ^a^	2613.30 ± 391.91 ^a^	1 3.35 ± 2.32	3.05 ± 0.51 ^a^	23.33 ± 3.18 ^ab^
CV	24.50	23.65	29.34	26.13	15.10	14.45	11.18
F-test	*	*	*	*	ns	**	*

Different letters indicate significant differences among values within the same column analyzed by the LSD test. Values are means ± SD (*n* = 4). * Significant difference at *p* ≤ 0.05; ** Significant difference at *p* ≤ 0.01; ns, non–significant difference.

**Table 4:** Tuber yield, number of tubers, tuber size, harvest index (HI), and tuber inulin contents of sunchoke at the harvest stage (120 days after planting) under field conditions.

Treatment	Yield Component		Weight of Tubers Per Plant (g)	Harvest Index (HI)	Inulin Contents (%)
	Number of Tubers Per Plant (Tuber)	Weight of Individual Tuber (g)			
T1: Control (no inoculation)	14.50 ± 1.73 ^c^	8.05 ± 1.45 ^c^	42.92 ± 6.28 ^c^	0.32 ± 0.11 ^c^	23.67 ± 2.16 ^c^
T2: Full dose of chemical fertilizers	18.25 ± 2.50 ^bc^	11.80 ± 4.16 ^abc^	70.23 ± 10.72 ^abc^	0.54 ± 0.07 ^ab^	30.48 ± 1.14 ^bc^
T3: Half dose of chemical fertilizers	18.00 ± 2.87 ^bc^	10.12 ± 0.87 ^bc^	65.72 ± 31.60 ^bc^	0.44 ± 0.06 ^bc^	33.27 ± 1.92 ^b^
T4: EPF inoculation	21.75 ± 0.96 ^ab^	13.20 ± 2.01 ^ab^	80.28 ± 22.69 ^ab^	0.55 ± 0.20 ^ab^	43.35 ± 4.46 ^a^
T5: AMF inoculation	22.75 ± 3.90 ^ab^	13.63 ± 1.52 ^ab^	87.64 ± 9.96 ^ab^	0.61 ± 0.19 ^ab^	46.30 ± 4.98 ^a^
T6: co-inoculation of EPF and AMF	25.75 ± 6.51 ^a^	14.33 ± 1.41 ^a^	98.33 ± 16.84 ^a^	0.64 ± 0.04 ^a^	50.32 ± 8.28 ^a^
CV	16.04	16.18	27.29	23.96	12.63
F-test	**	**	*	*	*

Different letters indicate significant differences among values within the same column by the LSD test. Values are means ± SD (*n* = 4). * Significant difference at *p* ≤ 0.05; ** Significant difference at *p* ≤ 0.01; ns, non-significant difference.

## Data Availability

Not applicable.

## References

[1] Yang L., He Q.S., Corscadden K., Udenigwe C.C. (2015). The prospects of Jerusalem artichoke in functional food ingredients and bioenergy production. Biotechnol. Rep..

[2] Qiu Y., Lei P., Zhang Y., Sha Y., Zhan Y., Xu Z., Li S., Xu H., Ouyang P. (2018). Recent advances in bio-based multi-products of agricultural Jerusalem artichoke resources. Biotechnol. Biofuels.

[3] Sennoi R., Singkham N., Jogloy S., Boonlue S., Saksirirat W., Kesmala T., Patanothai A. (2013). Biological control of southern stem rot caused by Sclerotium rolfsii using Trichoderma harzianum and arbuscular mycorrhizal fungi on Jerusalem artichoke (*Helianthus tuberosus* L.). Crop Prot..

[4] Nacoon S., Jogloy S., Riddech N., Mongkolthanaruk W., Ekprasert J., Cooper J., Boonlue S. (2021). Combination of arbuscular mycorrhizal fungi and phosphate solubilizing bacteria on growth and production of *Helianthus tuberosus* under field condition. Sci. Rep..

[5] Khaekhum S., Lumyong S., Kuyper T., Boonlue S. (2017). Species richness and composition of arbuscular mycorrhizal fungi occurring on eucalypt trees (*Eucalyptus camaldulensis* Dehnh.) in rainy and dry season. Curr. Res. Environ. Appl. Mycol..

[6] Nacoon S., Ekprasert J., Riddech N., Mongkolthanaruk W., Jogloy S., Vorasoot N., Cooper J., Boonlue S. (2021). Growth enhancement of sunchoke by arbuscular mycorrhizal fungi under drought condition. Rhizosphere.

[7] Suebrasri T., Somteds A., Harada H., Kanokmedhakul S., Jogloy S., Ekprasert J., Lumyong S., Boonlue S. (2020). Novel endophytic fungi with fungicidal metabolites suppress sclerotium disease. Rhizosphere.

[8] Khaekhum S., Ekprasert J., Suebrasri T., Mongkolthanaruk W., Riddech N., Jogloy S., Boonlue S. (2021). The first member of *Exserohilum rostratum* beneficial for promoting growth and yield of sunchoke (*Helianthus tuberosus* L.). Rhizosphere.

[9] Suebrasri T., Harada H., Jogloy S., Ekprasert J., Boonlue S. (2020). Auxin-producing fungal endophytes promote growth of sunchoke. Rhizosphere.

[10] Wężowicz K., Rozpądek P., Turnau K. (2017). Interactions of arbuscular mycorrhizal and endophytic fungi improve seedling survival and growth in post-mining waste. Mycorrhiza.

[11] Bilal L., Asaf S., Hamayun M., Gul H., Iqbal A., Ullah I., Lee I.-J., Hussain A. (2018). Plant growth promoting endophytic fungi *Asprgillus fumigatus* TS1 and *Fusarium proliferatum* BRL1 produce gibberellins and regulates plant endogenous hormones. Symbiosis.

[12] Liu H., Wu M., Liu J., Qu Y., Gao Y., Ren A. (2020). Tripartite interactions between endophytic fungi, arbuscular mycorrhizal fungi, and *Leymus chinensis*. Microb. Ecol..

[13] Van der Heijden M.G., Martin F.M., Selosse M.A., Sanders I.R. (2015). Mycorrhizal ecology and evolution: The past, the present, and the future. New Phytol..

[14] Klinsukon C., Lumyong S., Kuyper T.W., Boonlue S. (2021). Colonization by arbuscular mycorrhizal fungi improves salinity tolerance of eucalyptus (*Eucalyptus camaldulensis*) seedlings. Sci. Rep..

[15] Pandey R.R., Loushambam S., Srivastava A.K. (2020). Arbuscular Mycorrhizal and Dark Septate Endophyte Fungal Associations in Two Dominant Ginger Species of Northeast India. Proc. Natl. Acad. Sci. India Sect. B Biol. Sci..

[16] Domka A.M., Rozpaądek P., Turnau K. (2019). Are fungal endophytes merely mycorrhizal copycats? The role of fungal endophytes in the adaptation of plants to metal toxicity. Front. Microbiol..

[17] Hardoim P.R., Van Overbeek L.S., Berg G., Pirttilä A.M., Compant S., Campisano A., Döring M., Sessitsch A. (2015). The hidden world within plants: Ecological and evolutionary considerations for defining functioning of microbial endophytes. Microbiol. Mol. Biol. Rev..

[18] Xu F.-J., Song S.-L., Ma C.-Y., Zhang W., Sun K., Tang M.-J., Xie X.-G., Fan K.-K., Dai C.-C. (2020). Endophytic fungus improves peanut drought resistance by reassembling the root-dwelling community of arbuscular mycorrhizal fungi. Fungal Ecol..

[19] Sennoi R., Jogloy S., Saksirirat W., Patanothai A. (2010). Pathogenicity test of *Sclerotium rolfsii*, a causal agent of Jerusalem Artichoke (*Helianthus tuberosus* L.) stem rot. Asian J. Plant Sci..

[20] Hepper C.M. (1979). Germination and growth of Glomus caledonius spores: The effects of inhibitors and nutrients. Soil Biol. Biochem..

[21] Boonlue S., Surapat W., Pukahuta C., Suwanarit P., Suwanarit A., Morinaga T. (2012). Diversity and efficiency of arbuscular mycorrhizal fungi in soils from organic chili (*Capsicum frutescens*) farms. Mycoscience.

[22] Trouvelot A., Kough J., Gianinazzi-Pearson V., Gianinazzi-Pearson V., Gianinazzi S. (1986). Mesure du taux de mycorhization VA d’un système radiculaire. Recherche de méthode d’estimation ayant une signification fonctionnelle. Physiological and Genetical Aspects of Mycorrhizae, Proceedings of the 1st European Symposium on Mycorrhizae, Dijon, France, 1–5 July 1985.

[23] Walkley A., Black I.A. (1934). An examination of the Degtjareff method for determining soil organic matter, and a proposed modification of the chromic acid titration method. Soil Sci..

[24] Jackson M. (1967). Soil Chemical Analysis Prentice.

[25] Schuman G., Stanley M., Knudsen D. (1973). Automated total nitrogen analysis of soil and plant samples. Soil Sci. Soc. Am. J..

[26] Hesse P. (1971). A Textbook of Soil Chemical Analysis.

[27] Bray R.H., Kurtz L.T. (1945). Determination of total, organic, and available forms of phosphorus in soils. Soil Sci..

[28] Murphy J., Riley J.P. (1962). A modified single solution method for the determination of phosphate in natural waters. Anal. Chim. Acta.

[29] Arnon D.I. (1949). Copper enzymes in isolated chloroplasts. Polyphenoloxidase in Beta vulgaris. Plant Physiol..

[30] Bremner J. (1965). Total nitrogen. Methods Soil Anal. Part 2 Chem. Microbiol. Prop..

[31] Twine J., Williams C. (1971). The determination of phosphorus in Kjeldahl digests of plant material by automatic analysis. Commun. Soil Sci. Plant Anal..

[32] Saengkanuk A., Nuchadomrong S., Jogloy S., Patanothai A., Srijaranai S. (2011). A simplified spectrophotometric method for the determination of inulin in Jerusalem artichoke (*Helianthus tuberosus* L.) tubers. Eur. Food Res. Technol..

[33] Koske R., Gemma J. (1989). A modified procedure for staining roots to detect VA mycorrhizas. Mycol. Res..

[34] Mehmood A., Hussain A., Irshad M., Hamayun M., Iqbal A., Khan N. (2019). In vitro production of IAA by endophytic fungus *Aspergillus awamori* and its growth promoting activities in Zea mays. Symbiosis.

[35] Wang Y., Zhao Y., Xue F., Nan X., Wang H., Hua D., Liu J., Yang L., Jiang L., Xiong B. (2020). Nutritional value, bioactivity, and application potential of Jerusalem artichoke (*Helianthus tuberosus* L.) as a neotype feed resource. Anim. Nutr..

[36] Rossini F., Provenzano M.E., Kuzmanović L., Ruggeri R. (2019). Jerusalem artichoke (*Helianthus tuberosus* L.): A versatile and sustainable crop for renewable energy production in Europe. Agronomy.

[37] Rahimi A., Siavash Moghaddam S., Ghiyasi M., Heydarzadeh S., Ghazizadeh K., Popović-Djordjević J. (2019). The influence of chemical, organic and biological fertilizers on agrobiological and antioxidant properties of Syrian Cephalaria (*Cephalaria syriaca* L.). Agriculture.

[38] Copetta A., Todeschini V., Massa N., Bona E., Berta G., Lingua G. (2021). Inoculation with arbuscular mycorrhizal fungi improves melon (*Cucumis melo*) fruit quality under field conditions and plant performance in both field and greenhouse. Plant Biosyst. Int. J. Deal. Asp. Plant Biol..

[39] Zhu X., Song F., Xu H. (2010). Influence of arbuscular mycorrhiza on lipid peroxidation and antioxidant enzyme activity of maize plants under temperature stress. Mycorrhiza.

[40] Wijesooriya W., Deshappriya N. (2016). An inoculum of endophytic fungi for improved growth of a traditional rice variety in Sri Lanka. Trop. Plant Res..

[41] Verbruggen E., van der Heijden M.G., Rillig M.C., Kiers E.T. (2013). Mycorrhizal fungal establishment in agricultural soils: Factors determining inoculation success. New Phytol..

[42] Chung Y.A., Jumpponen A., Rudgers J.A. (2019). Divergence in diversity and composition of root-associated fungi between greenhouse and field studies in a semiarid grassland. Microb. Ecol..

[43] Giovannini L., Palla M., Agnolucci M., Avio L., Sbrana C., Turrini A., Giovannetti M. (2020). Arbuscular mycorrhizal fungi and associated microbiota as plant biostimulants: Research strategies for the selection of the best performing inocula. Agronomy.

[44] Rosa D., Pogiatzis A., Bowen P., Kokkoris V., Richards A., Holland T., Hart M. (2020). Performance and Establishment of a Commercial Mycorrhizal Inoculant in Viticulture. Agriculture.

[45] Aroca R., Ruiz-Lozano J.M., Zamarreño Á.M., Paz J.A., García-Mina J.M., Pozo M.J., López-Ráez J.A. (2013). Arbuscular mycorrhizal symbiosis influences strigolactone production under salinity and alleviates salt stress in lettuce plants. J. Plant Physiol..

[46] De Oliveira J., Rodrigues C., Vandenberghe L.P., Câmara M.C., Libardi N., Soccol C.R. (2017). Gibberellic acid production by different fermentation systems using citric pulp as substrate/support. BioMed Res. Int..

[47] Ibiang S.R., Sakamoto K., Kuwahara N. (2020). Performance of tomato and lettuce to arbuscular mycorrhizal fungi and *Penicillium pinophilum* EU0013 inoculation varies with soil, culture media of inoculum, and fungal consortium composition. Rhizosphere.

[48] Liu Q., Parsons A.J., Xue H., Fraser K., Ryan G.D., Newman J.A., Rasmussen S. (2011). Competition between foliar *Neotyphodium lolii* endophytes and mycorrhizal *Glomus* spp. fungi in Lolium perenne depends on resource supply and host carbohydrate content. Funct. Ecol..

[49] Roumeliotis E., Visser R.G., Bachem C.W. (2012). A crosstalk of auxin and GA during tuber development. Plant Signal. Behav..

[50] Javanmardi J., Rasuli F. (2017). Potato yield and tuber quality as affected by gibberellic acid and zinc sulfate. Iran Agric. Res..

[51] Shuab R., Lone R., Naidu J., Sharma V., Imtiyaz S., Koul K. (2014). Benefits of inoculation of arbuscular mycorrhizal fungi on growth and development of onion (*Allium cepa*) plant. Am.-Eurasian J. Agric. Environ. Sci..

